# Cytotoxic Activity of Antineoplastic Agents on Fertility: A Systematic Review

**DOI:** 10.1055/s-0040-1713911

**Published:** 2020-11-30

**Authors:** Gabriel Acácio de Moura, Paula Bruno Monteiro

**Affiliations:** 1 Universidade Estadual do Ceará, Fortaleza, CE, Brazil; 2 Centro Universitário Christus Unichristus, Fortaleza, CE, Brazil

**Keywords:** oncofertility, chemotherapy, radiotherapy, neoplasm, germ tissue, oncofertilidade, quimioterapia, radioterapia, neoplasia, tecidos germinativos

## Abstract

**Objective** To analyze the long-term effects of antineoplastic treatments on patient fertility.

**Selection of Studies** The studies were selected through the New PubMed, Scielo and Lilacs databases along with references used for the creation of the present work. For the selection of studies, articles published between the periods from January 1, 2015 to April 6, 2020 in the English, Portuguese and Spanish languages were used. As inclusion criteria: cohort studies and studies conducted in vitro. As exclusion criteria: review articles, reported cases, studies that do not address thematic reproduction, studies that do not address the cancer theme, articles that used animals, articles that address the preservation of fertility and articles in duplicate in the bases.

**Data Collection** The collected data included: age of the patient at the beginning of treatment, type of neoplasm, type of antineoplastic treatment, chemotherapy used, radiotherapy dosage, radiotherapy site, effect of antineoplastic agents on fertility and number of patients in the study.

**Data Synthesis** Thirty studies were evaluated, antineoplastic chemotherapy agents and radiotherapy modulate serum hormone levels, reduces germ cell quantities and correlated with an increase in sterility rates. The effects mentioned occur in patients in the prepubertal and postpubertal age.

**Conclusion** Antineoplastic treatments have cytotoxic effects on the germ cells leading to hormonal modulation, and pubertal status does not interfere with the cytotoxic action of therapies.

## Resumo

**Objetivo** Analisar os efeitos a longo prazo dos tratamentos antineoplásicos na fertilidade de pacientes.

**Fontes de dados** Os estudos foram selecionados através das bases de dados New PubMed, Scielo e Lilacs, junto com as referências utilizadas para a confecção do trabalho.

**Seleção dos estudos** Para a seleção dos estudos, foram utilizados artigos publicados entre os períodos de 01 de janeiro de 2015 a 06 de abril de 2020 nos idiomas inglês, português e espanhol. Como critérios de inclusão: estudos de coorte e estudos realizados in vitro. Como critérios de exclusão: artigos de revisão, relatos de caso, estudos que não abordavam a temática reprodução, estudos que não abordavam a temática câncer, artigos utilizando animais, artigos que abordavam preservação da fertilidade e artigos em duplicidade nas bases.

**Coleta de dados** Os dados coletados incluíram: idade do paciente ao início do tratamento, tipo de neoplasia, tipo de tratamento antineoplásico, quimioterápicos utilizados, dosagem da radioterapia, local da radioterapia, efeito dos agentes antineoplásicos na fertilidade e número de pacientes dentro do estudo.

**Síntese de dados** Trinta estudos foram avaliados. Os agentes quimioterápicos antineoplásicos e a radioterapia modulam níveis séricos hormonais de marcadores de fertilidade, reduzem a quantidade de células germinativas e estão correlacionados com um aumento da taxa de esterilidade. Os efeitos citados anteriormente ocorreram em pacientes com idade pré-púbere e pós-púbere.

**Conclusão** Os tratamentos antineoplásicos possuem efeitos citotóxicos em células germinativas, levando a modulação hormonal, e o status puberal não interfere diretamente na ação citotóxica das terapias.

## Introduction

Cancer (CA) is defined as a heterogeneous syndrome that evolves in several ways, this change being the result of a mutation in the activity of multiple oncogenes and tumor suppressor genes. 1 In this pathology, we can find much more than the proliferation of cancerous cell masses as well as the formation of complex cell phenotypes and distinct cell compounds participating in heterotypic interactions with each other. 2 According to the World Health Organization (WHO) in 2018, 3 CA was considered the main cause of death in the world, registering ∼ 9,6 million notifications.

The monitoring of the personal history of patients with CA is an object of great importance, since the increased risk of neoplasms may be directly associated with epidemiological risk factors such as cigarette use, alcoholism, obesity or genetic factors as predisposition to breast CA by the presence of the *BRCA1* gene and translocation of the *BCR-ABL* gene to leukemias. 4 Because of this, the frequent rush to develop methodologies that promote improvement in the survival of these patients has made great leaps. 5

Among the currently available therapeutic modalities, we can mention chemotherapy and radiotherapy, which cause several short-term effects accompanied by more serious adverse effects, such as cardiovascular activities, worsening renal function and nephrotoxicity. 6 7 8 In view of the above, therapies that aim to improve the chances of survival of the patient may end up having a significant weight, reducing their quality of life. 5

Antineoplastic chemotherapy agents (AQAs) are characterized by the use of cytotoxic drugs individually or in combination, to act in different phases of cell division, in cells that present dysfunction in the process of growth or cell division. 9 However, in the long run, due to the lack of specificity of AQAs, they can attack normal cells that renew themselves in regular periods as germ cells, having an important weight due to the high testicular and ovarian sensitivity to these cytotoxic agents. 10

Antineoplastic Radiotherapy (AR) consists of the administration of radioactive sources in preconditioned dosages on a body surface that leads to cell apoptosis of the cells in the region. 11 In women, pelvic radiotherapy in dosages established between 5 and 10 Gy is already considered toxic due to ovarian radiosensitivity. 10 In men, small direct doses as in 0.1 Gy therapy can cause damage to the sperm, leading to a drop in the number of sperms in the ejaculation. 12

The damage to the germinal organs depends on several factors, including the drugs used during treatment, the radiotherapy dose used, the age of the patient at the time of treatment and the basal state of the tissue, being used for the identification that caused loss of quality in the presence of a biomarker. 13 Thus, a hormonal dosage ends up becoming a valid alternative for the identification and prediction of the fertility of the patients, since these hormonal adjustments occur through neuroendocrine processes characterized by the activation of the hypothalamic pituitary-axis and release of luteinizing hormone (LH) and follicle stimulating hormone (FSH), for the development of both sexual male and female gametes, in addition to the presence of other markers such as the anti-mullerian hormone (AMH), estradiol (E _2_ ) and the antral follicles count (AFC), which mark sexual development. 14 15

Due to the different chronological rhythm of patients in prepubertal status, characterized by low dosages of hormonal release and absence of gamete development, it was believed that this profile of patients undergoing antineoplastic therapies reduced cytotoxic damage to germ cells. 16 However, studies on rats have shown that with antineoplastic treatment, rats in the prepubertal state have had their reproductive functions reduced or impaired. 17 18 19

Therefore, the present review aims to analyze, through a systematic review of the literature, the activity of antineoplastic treatments (chemotherapy and radiotherapy) on the fertility of patients undergoing CA treatment.

## Methods

### Study Type

The present study is a systematic literature review in which we outline an objective search methodology to answer the question regarding the proposed theme, using a collection of studies. 20 The present systematic review was performed according to the Preferred Reporting Items for Systematic Reviews and Meta-Analyses (PRISMA) guide reports ( http://www.prisma-statement.org/PRISMAStatement/ ). 21

### Study Eligibility Criteria

1 - Studies: Clinical trials were used for the present review, verifying different toxic effects of chemotherapy and radiotherapy or chemotherapy used alone in fertility;
2 - Publication period: Articles published in the period between January 1, 2015 and April 6, 2020 were considered eligible for analysis;
3 - Language of studies: Studies in English and Spanish were used to make the present work;
4 - Intervention used: Clinical trials involving chemotherapy and radiotherapy or just chemotherapy showing their adverse effects both in vivo and in vitro. Studies that address adverse unit effects of antineoplastic treatments with the aid molecules that would preserve fertility were disagreed studies.
5 - Outcomes: Effects of antineoplastic treatments on post-treatment patient gestational rate, effects of ovarian reserve of female patients through the measurement of hormonal markers such as HMA, E _2_ , LH and FSH; the same also applies to the production of sperm in male patients and defects in its morphology, quantity and viability.

### Search of Databases

For the search, the New PubMed, Scielo and Lilacs databases were used, and the search was performed manually. For its realization, a combination of advanced research was used among the descriptors for all databases: ( *Cancer* AND *Neoplasm* AND *Chemotherapy* *antineoplastic* AND *fertility* AND *effect* ) for chemotherapy. For radiotherapy, the following descriptors were used ( *Cancer* AND *Neoplasm* AND *Radiotherapy* *antineoplastic* AND *fertility* AND *effect* ).

## Selection Criteria for Studies

### Inclusion Criteria

1 - Cohort studies through clinical trials that analyzed the relationship between antineoplastic treatments and fertility;
2 - In vitro studies using cryopreservation and in vitro maturation of patients with CA.

### Exclusion Criteria

1 - Review articles;
2 - Case reports;
3 - Studies that did not address the theme of reproduction;
4 - Studies that did not address the theme of CA;
5 - Animal studies;
6 - Articles that addressed fertility preservation;
7 - Duplicate articles.

### Data Collection

Initially, a search was performed in the databases using the referring descriptors. Soon after, titles and abstracts of the articles were evaluated based on the aforementioned inclusion and exclusion criteria. Then, after selecting eligible articles for the creation of the present review, they were released for data extraction.

### Antineoplastic Chemotherapy

1 - Age of the patient at the diagnosis of CA;
2 - Type of neoplasm;
3 - Type of treatment (Chemotherapy + Radiotherapy or Chemotherapy alone);
4 - AQAs cited in the study;
5 - Possible AQA activity in fertility;
6 - Number of patients evaluated in the study.

### Antineoplastic Radiotherapy

1 - Age of the patient at the diagnosis of CA;
2 - Type of neoplasm;
3 - Type of treatment (Radiotherapy + Chemotherapy or Radiotherapy alone);
4 - Radiation site;
5 - Possible Antineoplastic Radiotherapy (AR) activity in fertility;
6 - Number of patients evaluated in the study;
7 - Dosage used.

## Results

**Fig. 1:**
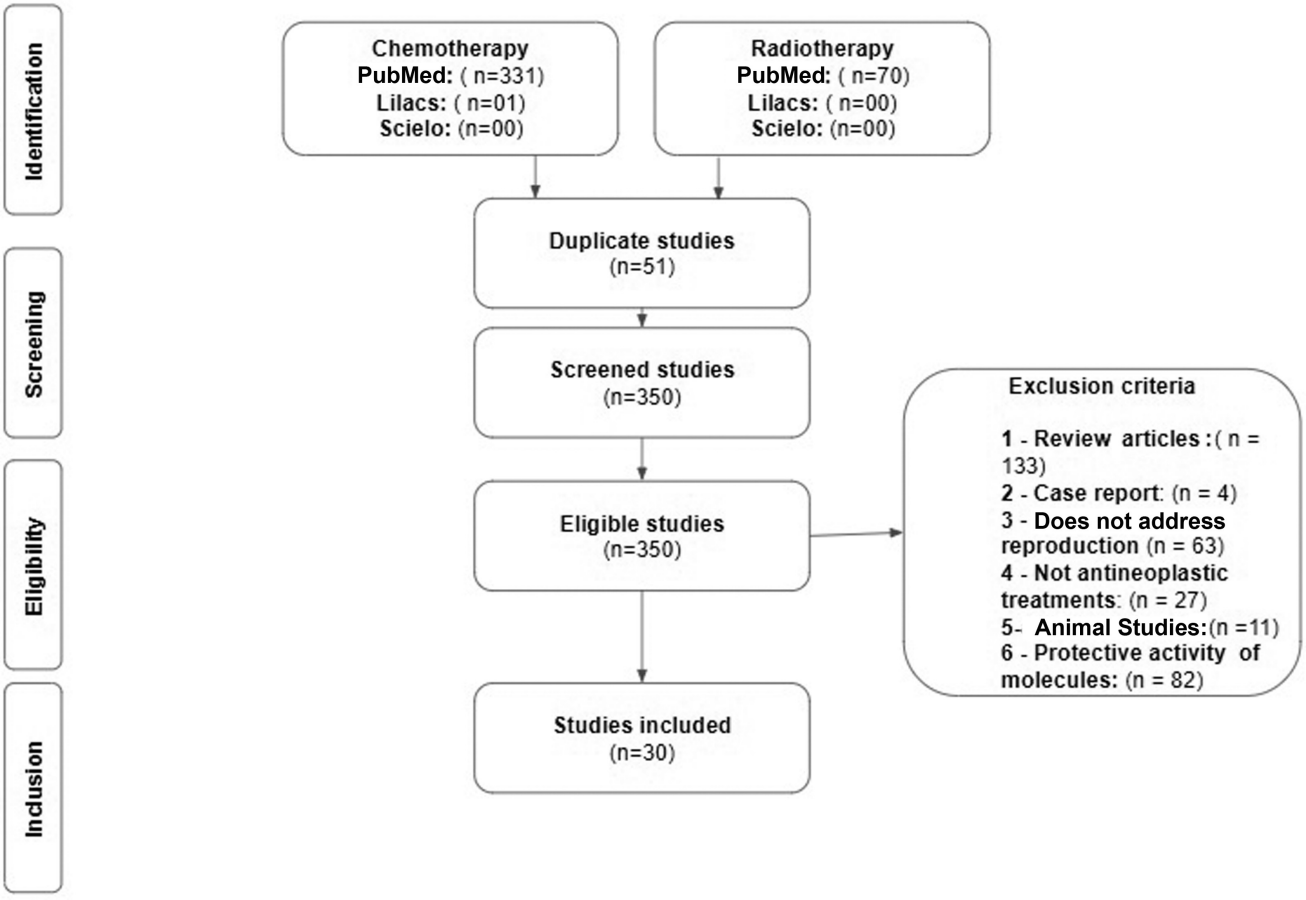
Methodological screening.

A total of 401 articles were found, of which 30 articles were selected from the screening in the New PubMed, Scielo and Lilacs databases for the elaboration of the present review, as shown in Fig. 1 .

In the present review, the literature demonstrated different antineoplastic therapies for different types of CA as show in Table 1 and Table 2 , with the main types of cancer studied being breast CA and Hodgkin Lymphoma. In these studies, the patients included had an age range from 0 to 50 years old.

**Table 1:** Influence of antineoplastic chemotherapy agents on fertility pattern

Author	Age	Tumor type	Treatment	Antineoplastic agent	Effect	Number of patients
Gini et al (2019) 22	16–50 years old	Hodgkin Lymphoma Non-Hodgkin Lymphoma	Chemotherapy + Radiotherapy	Etoposide Doxorubicin Cyclophosphamide Vincristine Prednisone Bleomycin	↑ Amenorrhea	97
Shandley et al (2018) 23	20–35 years old	Breast Cancer Lymphomas Reproductive Tumor	Chemotherapy	—	↓ AFC ↓AMH	1090
Sinha et al (2018) 24	24–43 years old	Breast Cancer	Chemotherapy	Taxotere Cyclophosphamide Carboplatin Fluorouracil Epirubicin	↓ AFC	88
Al-Rawi et al (2018) 25	25–45 years old	Breast Cancer	Chemotherapy	Anthracycline Cyclophosphamide	↓AMH ↓E _2_ ↑ LH	58
Anderson et al (2018) 26	18–45 years old	Hodgkin Lymphoma	Chemotherapy	—	↓AMH ↓E _2_ ↑ FSH	67
D'Avila et al (2017) 27	27–40 years old	Breast Cancer	Chemotherapy	Cyclophosphamide	↑ Amenorrhea ↑ FSH ↓AMH ↓ AFC ↑E _2_	49
Chang et al (2017) 28	15–51 years old	Chronic Myeloid Leukemia	Chemotherapy	Imatinib	↓ ejaculated volume ↓Sperm ↓ seminal density ↓ Viability Sperm	48
Creux et al (2017) 29	16–40 years old	Breast Cancer Cervical Cancer Hematological Cancer Gynecological Cancer Sarcoma Gastrointestinal Cancer	Chemotherapy	—	IVM - (normality)	164
Wenners et al (2017) 30	28–46 years old	Breast Cancer	Chemotherapy	Docetaxel Doxorubicin Fluorouracil Cyclophosphamide Epirubicin Paclitaxel Anthracycline	↓ ACF ↓AMH ↑ FSH ↑ LH	51
Perdrix et al (2017) 31	11–35 years old	Breast Cancer	Chemotherapy + Radiotherapy	Fluorouracil Epirubicin Cyclophosphamide Docetaxel	↓AMH	5775
McLaughlin et al (2017) 32	12–30 years old	Hodgkin Lymphoma	Chemotherapy	Adriamicina Bleomycin Vinblastine Dacarbazine Vincristine Etoposide Prednisone Doxorubicin Cyclophosphamide	↓ Follicular density ↓ Follicular development	13
Abir et al (2016) 33	5–18 years old	Medulloblastoma Acute Myeloid Leukemia Rhabdomyosarcoma Ewing Sarcoma Hodgkin Lymphoma Osteosarcoma	Chemotherapy	—	↑ Number of atretic follicles ↓ Oocyte maturation	20
Hamy et al (2016) 34	26–43 years old	Breast Cancer	Chemotherapy	—	↓AMH	134
Paoli et al (2016) 35	13–51 years old	Hodgkin Lymphoma	Chemotherapy	Doxorubicin Bleomycin Vinblastine Dacarbazine	↓Sperm ↑ Sperm anomalies	810
Even-Or et al (2016) 36	13 -36 years old	Acute Myeloid Leukemia	Chemotherapy	Melfalano	↓AMH ↓ LH ↓FSH	35
Jacobson et al (2016) 37	20–35 years old	Breast Cancer Acute Myeloid Leukemia Non Hodgkin Lymphoma	Chemotherapy + Radiotherapy	—	↑ Amenorrhea	1282
Gupta et al (2016) 38	11–18 years old	Osteosarcoma Hodgkin Lymphoma B cell Lymphoma Dysgerminoma Osteosarcoma	Chemotherapy	Cyclophosphamide Doxorubicin Cisplatine	↓AMH	17
Ruddy et al (2015) 39	40–45 years old	Breast Cancer	Chemotherapy	Trastuzumab Paclitaxel	↑ Amenorrhea	410
Huser et al (2015) 40	18–40 years old	Hodgkin Lymphoma	Chemotherapy	Adriamycin Bleomycin Vinblastine Vincristine Procarbazine Prednisone Etoposide Doxorubicin Cyclophosphamide	↑ FSH	108
Levi et al (2015) 41	20–44 years old	Colon Cancer Esophageal Cancer Thymic Cancer Rectal Cancer Neck Cancer	Chemotherapy	Cisplatine Capecitabine Fluoropyrimidine	↑ FSH ↓Inhibin B ↑ AMH	20
Thomas-Teinturier et al (2015) 42	18–39 years old	Bone Sarcoma Soft Tissue Sarcoma Neuroblastoma Hodgkin Lymphoma Non Hodgkin Lymphoma Acute Lymphoid Leukemia	Chemotherapy + Radiotherapy	Cyclophosphamide Ifosfamide Procarbazine busulfan	↑ FSH ↓AMH	105
Meissner et al (2015) 43	18–40 years old	Non Hodgkin Lymphoma	Chemotherapy	Cyclophosphamide Doxorubicin Vincristine Prednisone	↑ Amenorrhea ↓AMH	46

Abbreviations: AFC, antral follicle count; AMH, anti-mullerian hormone; E _2_ , estradiol; FSH, follicle stimulating hormone; IVM, in vitro maturation; LH, luteinizing hormone.

### Antineoplastic Chemotherapy Agents Modulate Serum Hormonal Secretion and Alter Germ Cell Production and Quality in Patients Undergoing Cancer Treatment

The literature shows us that, in different types of CA, the treatment with AQAs led to a modulation in serum hormonal concentrations. In 11 studies, there was a significant reduction in the hormonal levels of AMH, E _2_ , LH, and FSH. 22 23 24 25 26 27 28 29 30 31 32 33 34 35 36 37 38 39 40 41 42 43 In contrast, 8 studies showed an increase in hormone levels during treatment, such as FSH, LH, AMH, and E _2._ 25 26 27 30 33 40 41 42

Through our review, we can see that the production of the germ cells like oocytes and sperm has been altered. In two studies performed in patients undergoing treatment with male AQA, a reduction in the concentrate of sperm in the ejaculation was identified, as well as a reduction in the ejaculation and in the sperm survival, in addition to verifying the appearance of anomalies in its forms. 28 35

In seven clinical trials performed on female patients, there was a reduction in AFC and an increase in amenorrhea in the patients. 22 24 27 30 36 39 43 In vitro assays were also considered for the analysis of the effects of AQA on female fertility; in two studies, there was an increase in follicular density in addition to presenting a greater amount of atretic follicle. 32 33 The results are available in Table 1 .

### Radiotherapy Combined with Chemotherapy can Modulate Serum Hormonal Secretion and Alter the Production of Germ Cells, in Addition to Directly Interfering with Their Depletion

During the present review, two studies performed on patients undergoing chemotherapy regimens combined with radiation therapy at dosages < 40 Gy in the lymph node region led to a serum reduction of the hormones FSH and LH in male patients and of the chorionic gonadotropin (Hcg) hormone in female patients. Two authors showed a significant reduction in sperm concentration and progressive motility in male patients. A single study performed in vitro showed that with the administration of chemotherapy in combination with radiotherapy in the subdiaphragmatic region with a dosage of 20 Gy led to a reduction in the success rates of oocytes In Vitro Maturation (IVM) in these patients. Two studies also showed the ability that combination of antineoplastic treatments in concentrations ranging from 20 to 30 Gy had in depleting the ovarian content leading to premature ovarian failure (POF) syndrome, receiving radiation in the pelvic, sacral, and lumbar region, in the entire spine, in the flank of the abdomen, in the inverted Y region, in the para-aortic, in the iliac region, in the bladder region, in the vaginal and in the lymphoid region. In another cohort study performed with patients of both genders undergoing chemotherapy plus radiotherapy, there was the possibility of an increase in sterility rates in the patients. 44 45 46 47 48 49 50 51 The results are available in Table 2 .

**Table 2:** Influence of radiotherapy + antineoplastic chemotherapy agents on fertility pattern

Author	Age	Tumor type	Treatment	Corporal site	Dosage	Effect	Number of patients
Duhil de Bénazé et al (2018) 44	7–18 years old	Dysgerminoma	Chemotherapy + Radiotherapy	Lymphatic chains	20 Gy	↑ HcG	48
Fernandez-Pineda et al (2018) 45	4–22 years old	Hodgkin Lymphoma	Chemotherapy + Radiotherapy	Pelvic	9–55 Gy	↑ POF	127
Rives et al (2017) 46	24–43 years old	Testicular Cancer	Chemotherapy + Radiotherapy	Lymphatic chains	25 Gy	↓Sperm ↓ Sperm motility	54
Green et al (2017) 47	0–15 years old	Acute Lymphoid Leukemia	Chemotherapy + Radiotherapy	Testicular Hypothalamic Pituitary	40 Gy	↓Sperm ↓ Sperm motility ↓LH ↓FSH	171
Chemaitilly et al (2017) 48	18–45 years old	Leukemia Lymphoma Central Nervous System Tumors Embryonic Tumors Bone Tissue Sarcoma Carcinomas	Chemotherapy + Radiotherapy	Pelvic Lumbar region Sacral Entire Spine Abdominal flank Para-aortic Iliac Bladder Vaginal Lymphoid	30 Gy	↑ POF	988
Chow et al (2016) 49	5–20 years old	Leukemia Central Nervous System Tumors Lymphoma Renal tumor Neuroblastoma Soft Tissue Tumor Bone tumor	Chemotherapy + Radiotherapy	Neck Chest Legs Arms	—	↑ Sterility rate	10938
Boltežar et al (2016) 50	18–40 years old	Hodgkin Lymphoma	Chemotherapy + Radiotherapy	Pelvic	24 Gy	↑ Amenorrhea	131
Sonigo et al (2016) 51	17–33 years old	Hodgkin Lymphoma Breast Cancer	Chemotherapy + Radiotherapy	Subdiaphragmatic	> 26 Gy	↓IVM	22

Abbreviations: FSH, follicle atimulating hormone; HcG, human chorionic gonadotropin; IVM, in vitro maturation; LH, luteinizing hormone; POF, premature ovarian failure.

## Discussion

The increase in the number of more aggressive antineoplastic treatments can lead to improvements in the survival rate of young patients diagnosed with CA. 52 However, these therapies can also cause infertility and sterility, where providers and patients tolerate these adverse effects with the ideal of survival being the sole objective. 53 With the development of current assisted reproduction technologies and hormonal restoration procedures, including in vitro fertilization and ovarian transplantation, the possibilities for preserving the fertility of the patients have been increased; however, they do not provide long-term solutions and leave pediatric patients with metastatic tumors without options. 54

Currently, among the main types of treatment, are traditional chemotherapy and radiotherapy, which may be correlated in the short and long terms with effects on fertility that end up aggravating the suffering of patients during treatment·. 6 It is important to note that for men and women of childbearing age, concerns about loss of fertility may end up influencing the choice of adherence to treatment regimens. 55 Thus, the systematic resolution of adverse effects that may occur during treatment becomes something necessary and, being aware of the antineoplastic pharmacological cytotoxicity, it is possible to improve decision-making regarding treatment adherence. 56

In the present review, it was possible to observe a serum hormonal modulation with the use of AQA. Such results have also been presented in the literature, as in the study by Huddart et al, 57 who observed that 367 patients exposed to therapies with AQA had increased levels of LH and FSH. These results were found again in the study by Madhu et al in 2016, 58 who found an increase in LH and FSH levels and a decrease in testosterone after an initial treatment period with AQA. This is mainly due to hormonal feedback driven by the hypothalamic-pituitary gonadal axis in which the synthesis of these sex steroids will control the production of mature gametes present during therapy and will trigger an increase in their concentrations. 59 60

In our review, we can see that mainly AMH was modulated. These results are present in the literature, as previously observed by Levi et al, 41 who in studies with mice mimicking chemotherapy antineoplastic treatment found that there was a reduction in AMH in 19 mice. Such modification is due to the reduction in the amount of antral follicles present in the ovarian reserve in women and the reduction of male sexual gametes, which due to the fact that AMH is a glycoprotein present in the germinal tissues that end up producing these hormones ends up having the levels negatively modulated until complete recovery and therapy is stopped, and can be considered a good marker of ovarian reserve for patients undergoing CA treatment. 61

Another important factor observed in the present review was an increase in atretic follicles, reduction in Antral Follicle Count (AFC) in female patients, effects also observed in the concentration of sperm and changes in seminal parameters of patients exposed to AQA. The literature infers this activity of AQA to a nonspecificity of these agents, in which drugs that must attack cells with a marked proliferative phenotypic profile start to attack cells of similar profile leading to adverse effects. 62 Such activity will lead to the attack of somatic and granula cells, interfering directly in folliculogenesis, leading to poor follicular development both in vivo and in vitro. 63

The current consensus for treatments of some types of localized CA involves the provision of AR regardless of the use of chemotherapy. 64 However, in the present review, no articles were found using radiotherapy alone as a treatment choice. We observed that in concentrations between 20 Gy to a range of 55 Gy in the different regions to which the ion beam is applied, they exercise activities modulating both the hormonal levels and leading to patient sterility. These results are similar to the study by Isaksson et al, 65 who observed that radiotherapy located in testicular cancer led to modulation of sex hormones, including reduction of inhibin B.

During the present review, it was not possible to observe differences between effects from chemotherapy and radiotherapy in patients of different age groups, due to obtaining similar effects in the studies. What again comes into consensus with the literature according to a study by Paoli et al, 66 it is observed that age is not considered a determining factor for the protection or decline of fertility, corroborating the hypothesis that the effects of antineoplastic therapies on fertility are dose dependent.

## Conclusion

In conclusion, antineoplastic treatments have a potential cytotoxic effect on the fertility of patients undergoing CA treatment. The age at which the patient starts treatment does not show to be a protective factor or an accelerator of effects on fertility; however, the accumulated dosage in tissues has a fundamental impact on the fertility of these patients.
